# Iron deficiency at birth and risk of hidden hearing loss in infants modification by socioeconomic status: mother-newborn cohort in Shenyang, China

**DOI:** 10.1186/s12889-024-18439-4

**Published:** 2024-04-03

**Authors:** Shuai Hao, Wei Song, Fanxue Kong, Xinxin Yue, Xinlei Meng, Hongyan Chen, Yunyan Han, Fei Yu

**Affiliations:** 1 Department of Otolaryngology, First Affiliated Hospital of China Medical University, No. 155, Nanjing North Street, Heping District, Shenyang, 110001 China; 2 School of Public Health, He University, Hunnan New District, No.66 Sishui Street, Shenyang, 110163 China; 3 Department of Nutrition and Food Hygiene, School of Public Health, China Medical University, Shenyang North New District, No.77 Puhe Road, Shenyang, 110122 China; 4 Center of Physical Examination, First Affiliated Hospital of Dalian Medical University, No. 193 Lianhe Road, Xigang District, Dalian, 116011 China; 5 School of Clinical Medicine, He University, Hunnan New District, No.66 Sishui Street, Shenyang, 110163 China; 6 Department of Nutrition and Food Hygiene, School of Public Health, Dalian Medical University, No.9 West Section of Lvshun South Road, Dalian, 116044 Lvshunkou District China

**Keywords:** Hidden hearing loss, Iron deficiency, Newborn, Socioeconomic status

## Abstract

**Objective:**

The diagnosis of hidden hearing loss (HHL) in calm state has not yet been determined, while the nutritional status is not involved in its pathogenic risk factors. In utero iron deficiency (ID) may delay auditory neural maturation in infants. We evaluated the association between ID and HHL as well as the modification effect of socioeconomic status (SES) on this association in newborns.

**Study design:**

We included 859 mother-newborns from the baseline of this observational northeast cohort. Data on exposure assessment included iron status [maternal hemoglobin (Hb) and neonatal heel prick serum ferritin (SF)] and SES (occupation, education and income). Auditory neural maturation was reflected by auditory brainstem response (ABR) testing and electrocochleography (ECochG).

**Results:**

Iron status and SES were independently and jointly associated with the prediction of neonatal HHL by logistic and linear regression model. The mediation effects were performed by Process. ID increased absolute latency wave V, interpeak latency (IPL) III-V, and summting potentials (SP) /action potentials (AP), which were combined as HHL. Low SES showed the highest risk of HHL and the highest levels of related parameters in ID newborns. Moreover, after Corona Virus Disease 2019 (COVID-19) were positive, preschool children who experience ID in neonatal period were more likely to suffer from otitis media with effusion (OME). High SES also showed similar risk effects.

**Conclusion:**

Both low and high SES may strengthen the risk of ID on neonatal HHL in Northeast China.

**Supplementary Information:**

The online version contains supplementary material available at 10.1186/s12889-024-18439-4.

## Introduction

Iron deficiency (ID) is the most common form of micronutrient deficiency, and ID affects more than 2 billion people around the world [1]. ID is the main cause of anemia. Anemia remains a major public health problem, especially for children and women in low- and middle-income countries. Although the prevalence of anemia among young children (56.5%) and women (40.4%) decreased from 2000–2009 to 2010–2018 in most countries [2], the age-standardized point prevalence of anemia was 23.18% (1.8 billion) worldwide in 2019 [3]. For preschool children and young women in the world, dietary ID was also in the top ten burden of diseases in a large data from 204 countries between 1990 and 2019 [3]. The difference between ID and iron deficiency anemia (IDA) lies in the degree [4]. In fact, the estimated prevalence of ID is nearly twice that of IDA worldwide [5], which is a neglected health problem.

As another neglected global public health problem, hearing loss (hearing threshold above 20 decibels) affects the quality of life of nearly 360 million adults [6] and 550 million children [7]. Hearing loss ranked third in the burden of disease from 204 countries between 1990 and 2019 [3]. The main causes of hearing loss include heredity, infection, noise exposure, aging and ototoxic drugs [6]. Surprisingly, WHO only lists severe prenatal iodine deficiency as the nutritional cause of hearing loss. However, the etiology of hearing loss caused by dietary patterns and nutritional imbalances has not been determined yet [8]. Through auditory brainstem responses (ABRs) testing, ID and IDA may delay auditory nerve maturation in newborns [9, 10] and infants [9, 11] to varying degrees.

In animal experiments [12–14], as maternal ID progresses to IDA during pregnancy and lactation, the brainstem and peripheral auditory receptors are damaged to varying degrees in the auditory conduction pathway. The most sensitive areas of damage are ribbon synapse of inner hair cell (IHC) of cochlea. The impaired ribbon synapse leads to the inactivation of auditory nerve fibers (ANF) with high threshold and low spontaneous discharge rate, reducing conduction velocity. Also, the axon diameter affects conduction velocity. Then there may be delayed myelination, demyelination, reduction of mature oligodendrocytes, hair cells, and spiral neurons, or axonal mutations. Hidden hearing loss (HHL) is a new concept of hearing impairment [15]. Patients show normal threshold of pure tone audiometry results, and lack of ability to process complex speech information and time domain coding function, especially in noisy environment, that is, the speech recognition rate decreases in noisy environment. Its pathogenesis involves cochlear synaptopathy, demyelinating lesions and hair cell dysfunction, but the nutritional status of the body is not involved in its pathogenic risk factors.

Noteworthily, only two references included in Pubmed involve the "gold standard" of HHL, but both standards are exposed to specific white noise, showing a statistically difference compared with the unexposed in electrocochleography (ECochG) related indicators [16, 17]. The standard of HHL in calm state is not involved, moreover the nutritional status of the body is not involved in the risk factors of HHL.

Therefore, we proposed a hypothesis that ID may be related to HHL. The following phenomenon suggested this possibility again. During the Corona Virus Disease 2019 (COVID-19) pandemic, on December 14, 2022, the Chinese government no longer checked the nucleic acid tests, and the number of positive people reached its peak on December 22 (6.94 million/day) in China. Then, from December 2022 to March 2023, the incidence of otitis media with effusion (OME) in our outpatient department increased significantly compared with the incidence in the same month before the epidemic. Further through medical record inquiry and telephone follow-up, it was found that most of the school-age children were born in our hospital (2013–2017), and their mothers partially showed ID when they were admitted to hospital for physical examination, but not IDA. Moreover, the neonatal hearing was normal during neonatal hearing screening, and it was speculated that they might be in a state of HHL (COVID-19 may be a severe stimulation or exposure). At the same time, socioeconomic status (SES) is related to children's brain and behavior development [18]. However, evidence of the association between iron status and the prevalence of HHL as well as the modification effect of SES on this association in newborns is scarce.

To sum up, the first step of this study was to analyze the association between iron status and ECochG related indicators [absolute latency of wave I, III, and V, interpeak latency (IPL) I-III, III-V, and I-V, summting potentials (SP)/action potentials (AP) ratio (wave Ш, V)]. The second step was to analyze whether SES played a moderating or mediating effect in the delayed auditory neural maturation by ID. The third step was to observe whether ID in neonatal period could aggravate hearing loss and delay recovery in school-age children with hearing loss and OME caused by one-time strong exposure (COVID-19 positive) (combined with the first-step effect index, judge whether they were in HHL status in neonatal period, and determine the standard index).

## Materials and methods

### Sample

We conducted a prospective cohort study on auditory neural maturation at birth among mother-newborns with different iron status and SES. The study was conducted at First Affiliated Hospital of China Medical University, in Shenyang, the capital of Liaoning Province in Northeast China, from November 2013 to May 2017. We choose pregnant women aged 19–35, single births, full-term newborns, without deformities. The information of demographic and clinical profiles hemoglobin (Hb), serum ferritin (SF), hearing screening came from Department of Obstetrics and Otolaryngology, First Affiliated Hospital of China Medical University. All protocols were approved by the medical ethics committee of China Medical University [approval number 62033008]. These enrolled pregnant women signed an information and consent form. Maternal inclusion criteria: the pregnant women were 19–35 years old from Northeast China, and enrolled in late pregnancy (32–36 weeks of pregnancy, monozygous). Maternal exclusion criteria: maternal disease history during pregnancy; early birth or overdue birth, low birth weight or macrosomia, perinatal birth injury, dystocia or history of asphyxia and hypoxia, epilepsy, febrile convulsion and other central nervous system diseases, congenital heart disease and other organic diseases after birth metabolic diseases; recurrent diarrhea, infection and other chronic diseases or acute diseases during examination. Offspring exclusion criteria: according to the evaluation criteria of infant growth and development, in infants of the same sex and age, the weight and height were beyond their mean ± 2 standard deviations (SD); anemia and high sensitivity C-reactive protein; hypothyroidism, hyperlead or zinc deficiency, active rickets and other nutritional diseases, genetic metabolic diseases. We did not accept pregnant women with IDA into the investigation and study, and suggested oral ferric salt treatment. For people who met the inclusion criteria but did not participate in the trial, they still underwent the same medical examination as the subjects; Because the subjects included in the cohort were pregnant women who have not reached the status of IDA, there was no clinical iron supplementation treatment plan. It was suggested to strengthen the diet rich in iron, which was easy to absorb. The sample size calculation was based on $n\frac{={\left({\mu }_{\alpha }\sqrt{2\overline{p }\overline{q} }+{\mu }_{\beta }\sqrt{{p}_{1}{q}_{1}+{p}_{0}{q}_{0}}\right)}^{2}}{{\left({P}_{1}-{P}_{0}\right)}^{2}}$. We selected ID as the exposure factor and HHL as the outcome indicator. Due to the lack of HHL examination data for newborns, mild hearing loss was used as the outcome for estimation. According to our previous study, the incidence of mild hearing loss (p_0_) in neonatal hearing screening was 10%, with an estimated RR of 2. The values of each indicator are: α = 0.05, μ_α_ = 1.96, β = 0.1, μ_β_ = 1.28, q0 = 0.90, p_0_ = 0.10, p1 = 0.20, q_1_ = 0.80, $\overline{p }$= (0.10 + 0.20)/2 = 0.15, $\overline{q }$= 1-$\overline{p }$= 0.85. Assuming a 20% loss to follow-up rate, this study required at least 185 participants in each group. The sample size of this study met the requirements. Thus, we excluded 236 participants including 213 participants who did not meet the inclusion criteria, 12 participants who did not completed hearing screening, 11 participants who hid data information. Base previously sample size calculation [10] and the number of covariates included in the final regression model, we assumed a ratio of 1:3 for ID (*n* = 206) and normal iron status (NIS, *n* = 653) in our subject population. As a result, 859 mother-newborns were included in the final analyses.

SES is a comprehensive measure, including income, education level and employment status, which has an important impact on people’s health and disease recovery. Because tertiles SES primarily establish childhood health status, and low family SES can lead to poor neurodevelopment of children [18]. We established a comprehensive scoring system of family SES, which is composed of the education and occupation level of each parent in the family, household income in the past year. Annual household income (unit: RMB) was divided into three grades and scored: low (< 10,000/month, 1 point), middle (10,000 ~ 16,000/month, 2 points) and high (16,000 ~ /month, 3 points), the income criteria was based on economic situation of Northeast China. Occupation was divided into three grades and scored: farmers, manual workers, unemployed people (low, 1 point), businessmen or employees (middle, 2 points), leaders, professionals, or government employees (high, 3 points). Education was divided into three grades and scored: lower than high school (low, 1 point), high school graduation or equivalent (middle, 2 points), university graduation or above (high, 3 points). Then we calculated the total SES scores (3–9 points), and divided families into approximate tertiles of SES status distribution: low (≤ 4 points), middle (5–7 points) and high (≥ 8 points).

### Outcome assessment: auditory neural maturation

The ABR and ECochG were performed and analyzed by the audiologists who skilled in administering electrophysiological measurements to newborns between 48 and 72 h after birth.

ABR was performed with GN Otometrics tester. These subjects slept naturally. We used IHS SmartEP auditory evoked potential meter to record in a soundproof and shielded room. The stimulus signal is a short sound with alternating polarity, with a pulse width of 0.1 ms and a repetition rate of 19.3 times/s. The recording electrode was placed in the forehead hairline, the reference electrode was placed in the ipsilateral mastoid, the contralateral mastoid was grounded, and the local skin was degreased with alcohol cotton ball before placing the electrode. Peripheral auditory sensitivity and nerve conduction function of brainstem auditory pathway were mainly detected. The inter pole resistance is less than 10 k Ω. Filter bandpass from 100 to 1500 Hz. We selected the ABR results of one ear with a shorter V-wave latency to measure the absolute latency of wave I, III, and V, as well as IPLs (I-III, III-V, and I-V waves).

ECochG recorded three kinds of potentials, namely AP and SP. The measurement used alternating polarity of short sound stimulation, tympanic membrane electrode. After cleaning the external auditory canal and tympanic membrane with acetone solution, the electrodes were placed on the surface of the posterior lower part of tympanic membrane for recording. Click stimuli (0.1 ms) of 70 dB nHL was presented at a rate of 7.1/s to the test ear through an insert earphone (ER-3A). Waveforms were collected, the stimulus response was passed through a bandpass filter with a range of 5 to 2000 Hz and take the average value. Then SP/AP was calculated. The above outcome assessments were considered as indicators of auditory nerve maturation at different levels of auditory pathways. Impaired auditory neural maturation was defined as high absolute latency wave V, IPL III-V and SP/AP ratio above the 50th percentile [19].

### Covariates

Through face-to-face surveys of mothers, we collected the demographics and life style of mothers [age, gestational age, hemoglobin (Hb), fasting blood glucose (FBG), body mass index (BMI), activities, smoking, drinking, dietary pattern, cesarean delivery, assisted reproductive technology, hypertension], newborns (SF, birth weight, Apgar score, sex), and children [different symptoms of COVID-19 (ever, some throat, earache, hearing loss, ear tightness, resolved sense of smell, nasal conference, false, muscle soreness, cough)].

### Statistical analyses

We used EpiData 4.0 software (EpiData Association, Denmark) to enter data in the form of double entry, and finally selected 10% of the questionnaires for review. SPSS 20.0 software (IBM SPSS, Inc., Chicago, IL, USA) was used for statistical analysis on the data.

The data of normal distribution were expressed as mean ± SD. The data of the skewed distribution (FBG) was expressed as median (interquartile, IQR: Q1-Q3) or as Logarithm transformation (LGFBG) for mean ± SD. The continuous variables with normal distribution were compared by using two-sample t-test between two groups (ID compared with NIS), by Scheffe test of analysis of variance (ANOVA) among iron status quartile (range) and tertiles SES. Other variables with skewed distribution were compared by using Mann–Whitney U test between two groups (ID compared with NIS) or by using Kruskal–Wallis H test among iron status quartile (range) through tertiles SES. For categorical variables, Chi-square test or Fisher’s exact test was used to compare frequency (percentage, %) of partial demographic characteristics. Pearson correlation and intraclass correlation coefficients (ICCs) were used to assess the variability and reproducibility of neonatal SF and maternal Hb by SES tertiles. Parametric Pearson and Non-parametric Spearman correlation coefficients were calculated between iron status and auditory neural maturation by SES.

A linear regression model was used to evaluate the independent associations of SES and iron status with auditory neural maturation-related parameters (absolute latency wave V, IPL III-V, and SP/AP ratio). Dependent variables included neonatal absolute latency wave V, IPL III-V, and SP/AP ratio (wave V). Independent variables included maternal Hb and neonatal SF. In the dose–response analyses, Hb and SF levels were categorized into four quartiles, the first quartile was assigned as reference. Also, Multiple linear regression model was used to evaluate the effect estimates [*β* and 95% confidence interval (CI)] of SES and iron status on HHL risk, respectively. Absolute latency wave V, IPL III-V, SP/AP ratio (wave V) were as separately dependent variables and iron status biomarkers (neonatal SF and maternal Hb) were as independent variables after controlling potential confounders.

Multiple logistic regression model was used to assess the effect estimates [odds ratio (OR) and 95% CI] of SES and ID on children OME, respectively. Children OME after COVID-19 was as dependent variable, and ID was as independent variables after controlling for Birth weight, cesarean delivery and assisted reproductive technology. Also, multiple logistic regression of relationship between possible HHL criteria in newborn as dependent variable and ID as independent variables after controlling above covariates.

To examine whether ID mediates a link between SES and HHL prevalence, mediation analyses were performed by using the addition of Process to SPSS. The combined effects of iron status and SES on OME risk and HHL potential diagnostic markers (higher wave V, IPL III-V, and SP/AP ratio) were examined by comparing six combinations of different SES (low, moderate, high) and iron status (ID and NIS). The combination of NIS and high SES was considered as the reference group. Children adenoid hypertrophy, chronic sinusitis and allergic rhinitis may affect their hearing functions, sensitivity analysis was carried out after excluding children with adenoid hypertrophy, chronic sinusitis and allergic rhinitis. In addition, COVID-19 was the main confounding factor of children OME, a sensitivity analysis was conducted by additionally adjusting different symptoms of COVID-19 (ever, some throat, earache, hearing loss, ear tightness, resolved sense of smell, nasal conference, false, muscle soreness, cough).

## Results

The 236 mother-newborn excluded only participated in antenatal examination, but were unwilling to disclose the information of neonatal hearing screening. Except for the index of SES, no significant difference in other characteristics of mother-newborn were observed between the subjects included and excluded. The subjects excluded had a higher proportion of high SES, with lower proportion of moderate SES (Table 1).

**Table 1:** Baseline characteristics of mother-newborn included and excluded

Baseline characteristics	Included (*n* = 859)	Excluded (*n* = 236)	*p*-Value
Maternal factors			
Age at delivery (years)	29.20 ± 5.13	29.43 ± 4.69	0.542
Gestational age (weeks)	39.39 ± 1.98	39.42 ± 1.82	0.837
In the third trimester of pregnancy			
Hb (g/L)	122.36 ± 13.10	120.53 ± 12.38	0.055
FBG (mmol/L) ^a^	4.50 (4.20–4.90)	4.50 (4.10–4.90)	0.517
Pre-pregnancy BMI (kg/m^2^)	22.74 ± 1.52	22.70 ± 1.58	0.785
SES			0.001
Low	298 (34.7)	80 (33.9)	
Moderate	304 (35.4)	52 (22.0)	
High	257 (29.9)	104 (44.1)	
Extra-sport activities during pregnancy	377 (43.9)	103 (43.6)	0.947
Smoking before pregnancy	7 (0.8)	3 (1.3)	0.457
Passive smoking during pregnancy	196 (22.8)	53 (22.5)	0.907
Drinking before pregnancy	84 (9.8)	31 (13.1)	0.136
Dietary pattern			0.875
Vegetarian diet	189 (22.0)	52 (22.0)	
Meat diet	280 (32.6)	73 (30.9)	
Mixed diet	390 (45.4)	111 (47.0)	
Cesarean delivery	498 (58.0)	141 (59.7)	0.625
Assisted reproductive technology	59 (6.9)	23 (9.7)	0.137
Hypertension during pregnancy	30 (3.5)	9 (3.8)	0.814
Neonatal factors			
Heel prick blood SF (ng/mL)	89.27 ± 25.47	91.36 ± 18.82	0.164
Birth weight	3491.80 ± 273.28	3464.70 ± 297.23	0.208
Apgar score at 5 min	9.99 ± 0.13	9.99 ± 0.11	0.907
Male sex	524 (61.0)	157 (66.5)	0.121

Data are shown as n (%) or mean ± SD

^a^Values are presented as median (Q1-Q3)

*FBG* Fasting blood glucose, *BMI* Body mass index, *Hb* Hemoglobin, *SF* Serum ferritin, *SES* Socioeconomic status

Of 859 subjects included, 206 (24.0%) mother-newborns were in ID and 653 (76.0%) were in NIS during pregnancy. The demographic and clinical characteristics of mother-newborn-child were showed in Table 2. Iron status (neonatal heel prick blood SF and maternal Hb) in ID were lower than those in NIS, respectively. The partial index reflecting auditory neural maturation [wave V, IPL III-V, SP/AP ratio (wave V)] in ID were higher than those in NIS, respectively. The children who were at risk of ID in utero had higher the incidence (9.1%) of OME after COVID-19, than those (3.2%) in NIS. From November 2013 to May 2017, 1059 subjects (mother-newborn) were enrolled in the cohort study. Supplementary Table 1 showed the differences in basic characteristics of 859 subjects included among three SES (low, moderate, and high). Iron status (neonatal heel prick blood SF and maternal Hb) in moderate SES were higher than those in low and high SES, respectively. But, 243 (81.5%) mother-newborns in low SES were ID, and were more than those in moderate and high SES. BMI (23.49 ± 1.57) and birth weight (3542.65 ± 244.61 g) in low SES were the highest among three SES. The children in moderate SES had the lowest incidence (96.2%) of COVID-19.

**Table 2:** Characteristics of mother-newborn-child with iron deficiency and normal iron status

Characteristics	ID (*n* = 206)	NIS (*n* = 653)	*p*-Value
**Mothers**			
Age at delivery (years)	29.26 ± 5.08	29.00 ± 5.27	0.526
Gestational age (wk)	39.40 ± 1.97	39.35 ± 2.03	0.737
In the third trimester of pregnancy			
Hb (g/L)	126.08 ± 12.52	110.54 ± 5.97	0.001
LGFBG (mmol/L)^*^	0.66 ± 0.57	0.66 ± 0.57	0.551
Pre-pregnancy BMI (kg/m^2^)	22.57 ± 1.48	22.79 ± 1.53	0.076
SES			0.016
Low	243 (37.2)	55 (26.7)	
Moderate	226 (34.6)	78 (37.9)	
High	184 (28.2)	73 (35.4)	
Extra-sport activities during pregnancy	288 (44.1)	89 (43.2)	0.820
Pre-pregnancy smoking	4 (1.5)	3 (0.6)	0.368
Passive smoking during pregnancy	145 (22.2)	51 (24.8)	0.447
Pre-pregnancy drinking	65 (10.0)	19 (9.2)	0.758
Dietary pattern			0.875
Vegetarian diet	147 (22.5)	42 (20.4)	
Meat diet	210 (32.2)	70 (34.0)	
Mixed diet	296 (45.3)	94 (45.6)	
Cesarean delivery	281 (43.0)	80 (38.8)	0.287
Assisted reproductive technology	44 (6.7)	15 (7.3)	0.788
Hypertension during pregnancy	24 (3.7)	6 (2.9)	0.603
**Newborns**			
Heel prick blood SF (ng/mL)	87.65 ± 27.27	94.42 ± 17.74	0.001
Birth weight (g)	3495.70 ± 271.97	3479.47 ± 277.67	0.458
Apgar score at 5 min	9.99 ± 0.14	9.99 ± 0.10	0.803
Male sex	400 (61.3)	124 (60.2)	0.785
Absolute latency (ms)			
Wave I	1.81 ± 0.30	1.78 ± 0.35	0.268
Wave III	4.27 ± 0.61	4.26 ± 0.57	0.719
Wave V	5.99 ± 0.84	5.81 ± 0.72	0.002
IPL (ms)			
LG I-III	0.39 ± 0.07	0.39 ± 0.07	0.745
LG III-V	0.35 ± 0.09	0.33 ± 0.07	0.001
LG I-V	0.67 ± 0.07	0.66 ± 0.07	0.133
SP/AP ratio (wave III)	27.13 ± 4.10	26.62 ± 4.08	0.118
SP/AP ratio (wave V)	27.99 ± 4.41	26.98 ± 3.76	0.003
**Children**^**a**^			
COVID-19	185 (97.4)	578 (98.5)	0.323
OME			0.019
No	178 (94.7)	517 (88.7)	
After COVID-19	6 (3.2)	53 (9.1)	
Before COVID-19	4 (2.1)	13 (2.2)	

Data are shown as n (%) or mean ± SD

^*^Logarithm transformation

^a^Total number has missing data *LG* Logarithm transformation, *AP* Action potential, *SP* Summating potential, *OME* Otitis media with effusion, *COVID-19* Corona Virus Disease 2019, *ID* Iron deficiency, *NIS* Normal iron status, *IPL* Interpeak latency, *FBG* Fasting blood glucose, *BMI* Body mass index, *Hb* Hemoglobin, *SF* Serum ferritin, *SES* Socioeconomic status

Distributions of neonatal SF and maternal Hb concentrations were shown in Supplementary Table 2. The SF and Hb of moderate SES were highest among tertiles SES. Pearson correlation coefficients of SF and Hb among three SES ranged from 0.26 to 0.66. The ICC values for SF and Hb measurements of 0.58 and 0.38, respectively. This revealed a higher level of reproducibility in SF compared to the Hb concentrations. Supplementary Table 3 showed Pearson correlation coefficients between neonatal SF, maternal Hb and absolute latency (wave V), SP/AP ratio (wave V), and Spearman correlation coefficients between neonatal SF, maternal Hb and IPL III-V. These values indicated that there was a moderate correlation between the average values of iron status and auditory neural maturation. All correlation coefficients between SF and auditory neural maturation were higher than those between Hb and auditory neural maturation.

Supplementary Table 4 showed the mean levels of absolute latency wave V by quartiles of neonatal SF and maternal Hb. Significant differences of absolute latency wave V among quartiles of the SF (*P* < 0.001) and Hb (*P* < 0.05) were observed in all newborns and tertiles SES. Supplementary Table 5 showed the median (*P*_25_-*P*_75_) levels of IPL III-V by quartiles of neonatal SF and maternal Hb. Significant differences of IPL III-V among quartiles of the SF (*P* < 0.001) and Hb (*P* < 0.05) were observed in all newborns and tertiles SES. Supplementary Table 6 showed the mean levels of SP/AP ratio (wave V) by quartiles of neonatal SF and maternal Hb. Significant differences of SP/AP ratio (wave V) among quartiles of the SF (*P* < 0.001) and Hb (*P* < 0.05) were observed in all newborns and tertiles SES.

Table 3 presented the associations between neonatal SF, maternal Hb and absolute latency wave V, IPL III-V, SP/AP ratio (wave V), respectively. After adjustment, SF and Hb concentrations were significantly negatively associated with these auditory-related parameters. The negative regression coefficients reduced significantly with the increasing of SF and Hb concentrations (*P* < 0.05 for all). The highest neonatal SF concentrations was associated with a worst SP/AP ratio (-5.912; 95% CI: -6.593, -5.231 for Q4 vs. Q1). Supplementary Tables 7, 8 and 9 presented the associations between neonatal SF, maternal Hb and absolute latency wave V, IPL III-V, SP/AP ratio (wave V) in low, moderate, high SES, respectively. After adjustment, the negative regression trends were similar to those in all subjects. The highest SF concentrations was associated with a worst SP/AP ratio (-5.887; 95% CI: -6.886, -4.888 for Q4 vs. Q1) in low, (-5.437; 95% CI: -6.653, -4.222 for Q4 vs. Q1) moderate, (-6.552; 95% CI: -7.974, -5.130 for Q4 vs. Q1) high SES, respectively. No statistically significant associations were observed between Hb concentrations and SP/AP ratio in low SES, between Hb and these auditory-related parameters in moderate SES, between Hb and IPL III-V, SP/AP ratio (wave V) in high SES, respectively.

**Table 3:** Associations between neonatal SF, maternal Hb and neonatal absolute latency wave V, IPL III-V, and SP/AP ratio (wave V)

Iron status		Absolute latency wave V		IPL III-V		SP/AP ratio	
	*n*	*β* (95% CI)	*P*	*β* (95% CI)	*P*	*β* (95% CI)	*P*
SF (ng/mL)							
Q1	216	Ref	< 0.001	Ref	< 0.001	Ref	< 0.001
Q2	211	-0.714 (-0.854, -0.574)		-0.072 (-0.086, -0.057)		-3.926 (-4.611, -3.241)	
Q3	216	-0.818 (-0.958, -0.679)		-0.091 (-0.105, -0.076)		-5.360 (-6.041, -4.679)	
Q4	246	-0.871 (-1.010, -0.732)		-0.106 (-0.121, -0.091)		-5.912 (-6.593, -5.231)	
Hb (g/L)							
Q1	216	Ref	0.002	Ref	< 0.001	Ref	0.013
Q2	211	-0.234 (-0.382, -0.086)		-0.030 (-0.046, -0.014)		-0.984 (-1.758, -0.210)	
Q3	216	-0.459 (-0.607, -0.311)		-0.050 (-0.066, -0.034)		-2.718 (-3.491, -1.945)	
Q4	246	-0.515 (-0.670, -0.360)		-0.061 (-0.078, -0.044)		-2.452 (-3.260, -1.643)	

*Hb* hemoglobin, *SF* Serum ferritin, *IPL* Interpeak latency

Tables 4, 5 and 6 showed the multiple linear regression of relationship between absolute latency wave V, IPL III-V, and SP/AP ratio (wave V) as separately dependent variable and iron status biomarkers (neonatal SF and maternal Hb) as independent variables after controlling potential confounders. Neonatal SF was a significant predictor of these indicators of auditory neural maturation in all SES (*P* < 0.001). Maternal Hb was a significant predictor of absolute latency wave V and IPL III-V in low and moderate SES (*P* < 0.05), but no negative association was found in high SES. Hb levels were not associated with SP/AP ratio in all SES. For absolute latency wave V, cesarean delivery exhibited significant negative association with the latency value in moderate SES, Birth weight also showed significant negative association in high SES (*P* < 0.05 for all). For IPL III-V, assisted reproductive technology showed negative association with the values of IPL in moderate SES (P < 0.05). For SP/AP ratio, none of potential factors presented the significant associations.

**Table 4:** Multiple linear regression model of iron status (newborn SF and maternal Hb) and absolute latency wave V (ms) by SES

	Low SES			Moderate SES			High SES		
	*β* (95% CI)	*P*	ad R^2^	*β* (95% CI)	*P*	ad R^2^	*β* (95% CI)	*P*	ad R^2^
For absolute latency wave V		< 0.001	0.171		0.021	0.169			0.234
SF	-0.340 (-0.343, -0.336)	< 0.001		-0.359 (-0.363, -0.356)	< 0.001		-0.419 (-0.423, -0.395)	< 0.001	
Hb (g/L)	-0.181 (-0.188, -0.173)	0.003		-0.340 (-0.130, -0.117)	0.029		-0.097 (-0.105, -0.089)	0.118	
Age at delivery (years)	0.006 (-0.018, 0.020)	0.912		-0.003 (-0.021, 0.014)	0.959		0.029 (0.010, 0.048)	0.625	
Gestational age (wk)	0.047 (0.043, 0.092)	0.392		-0.004 (-0.047, 0.039)	0.942		-0.045 (-0.093, 0.002)	0.427	
Pre-pregnancy BMI (kg/m^2^)	0.059 (-0.005, 0.124)	0.341		0.014 (-0.050, 0.078)	0.804		0.015 (-0.048, 0.079)	0.798	
LGFBG (mmol/L) ^a^	0.026 (-0.108, 0.165)	0.630		0.040 (-0.083, 0.184)	0.459		-0.043 ( -0.197, 0.094)	0.444	
Extra-sport activities during pregnancy	0.030 (-0.160, 0.219)	0.594		-0.004 (-0.176, 0.169)	0.941		-0.001 ( -0.199, 0.197)	0.985	
Smoking before pregnancy	0.079 (0.317, 2.145)	0.160		0.069 (-0.673, 0.912)	0.203		0.066 (-1.447, 1.578)	0.247	
Passive smoking during pregnancy	-0.090 (-0.446, -0.041)	0.106		-0.009 (-0.219, 0.201)	0.872		-0.001 (-0.234, 0.233)	0.996	
Drinking before pregnancy	0.025 (-0.306, 0.355)	0.657		-0.031 (-0.317, 0.255)	0.563		0.022 (-0.270, 0.313)	0.707	
Mixed diet	0.011(-0.101, 0.123)	0.847		-0.090 (-0.193, 0.012)	0.092		0.088 ( -0.017, 0.226)	0.119	
Cesarean delivery	0.062(-0.043, 0.339)	0.279		-0.120 (-0.298, -0.032)	0.038		0.013 (-0.172, 0.237)	0.828	
Assisted reproductive technology	0.083(-0.223, 0.478)	0.128		-0.032 (-0.362, 0.298)	0.556		0.071(0.016, 0.694)	0.218	
Hypertension during pregnancy	0.023 (-0.346, 0.392)	0.681		0.195 (-0.111, 0.985)	0.386		-0.077 ( -0.837, 0.683)	0.175	
Birth weight	0.039 (0.037, 0.044)	0.506		-0.066 (-0.069,-0.065)	0.235		0.128 (0.127, 0.130)	0.027	
Apgar score at 5 min	-0.040 (-0.724, 0.644)	0.462		-0.028 ( -0.465, 0.402)	0.622		-0.096 (-1.566, 1.394)	0.090	
Female	0.028 (-0.117, 0.253)	0.610		0.090 (-0.158,0.176)	0.091		0.007 (-0.175, 0.208)	0.905	

^a^ *LG* logarithm transformation, *SES *Socioeconomic status, *FBG *Fasting blood glucose, *BMI *Body mass index, *Hb *Hemoglobin, *SF *Serum ferritin

**Table 5:** Multiple linear regression model of iron status (newborn SF and maternal Hb) and IPL III-V (ms) by SES

Multivariate regression	All		Low SES		Moderate SES		High SES	
	OR (95% CI)	*P*	OR (95% CI)	*P*	OR (95% CI)	*P*	OR (95% CI)	*P*
For high absolute latency wave V (> 10%)								
ID	1.214 (1.379, 0.702)	0.489	1.263 (0.481, 3.319)	0.636	1.580 (0.617, 4.049)	0.340	1.357 (0.515, 3.571)	0.536
Cesarean delivery	1.130 (0.721, 1.771)	0.594	2.153 (0.981, 4.723)	0.056	1.393 (0.651, 2.976)	0.393	1.002 (0.433, 2.319)	0.997
Birth weight	1.000 (1.000, 1.001)	0.331	1.001 (0.999, 1.002)	0.314	1.000 (0.998, 1.001)	0.490	1.001 (1.000, 1.003)	0.118
Assisted reproductive technology	2.080 (0.635, 6.809)	0.226	–-	–-	1.078 (0.234, 4.972)	0.923	2.308 (0.292, 18.230)	0.428
High absolute latency wave V (> 25%)								
ID	1.204 (0.844, 1.718)	0.305	1.886 (0.994, 3.581)	0.052	1.164 (0.633, 2.141)	0.626	1.160 (0.617, 2.183)	0645
Cesarean delivery	1.020 (0.755, 1.400)	0.899	1.561 (0.909, 2.692	0.106	1.383 (0.816, 2.342)	0.228	1.115 (0.618, 2.012)	0.717
Birth weight	1.000 (0.999, 1.000)	0.704	1.001 (1.000, 1.002)	0.090	0.999 (0.998, 1.000)	0.018	1.000 (0.999, 1.001)	0.699
Assisted reproductive technology	1.949 (0.904, 4.042)	0.073	2.199 (0.478, 10.111)	0.311	1.259 (0.435, 3.64)	0.671	3.216(0.723, 14.302)	0.125
High absolute latency wave V (> 50%)								
ID	1.680 (1.220, 2.313)	0.001	2.408 (1.295, 4.476)	0.005	1.293 (0.757, 2.207)	0.347	1.984 (1.117, 3.524)	0.019
Cesarean delivery	1.164 (0.883, 1.400)	0.286	1.213 (0.754, 1.951)	0.425	1.700 (1.070, 2.700)	0.025	1.067 (0.645, 1.767)	0.799
Birth weight	1.000 (0.999, 1.000)	0.254	1.000 (0.999, 1.001)	0.914	0.999 (0.998, 1.000)	0.047	1.000 (0.999, 1.001)	0.945
Assisted reproductive technology	1.949 (0.904, 4.042)	0.073	2.184 (0.726, 6.566)	0.164	1.220 (0.487, 3.056)	0.672	2.358 (0.881, 6.309)	0.088
For high IPL III-V (> 10%)								
ID	1.285 (0.735, 2.247)	0.379	1.012 (0.360, 2.840)	0.982	2.717 (0.882, 8.403)	0.082	1.056 (0.442, 2.525)	0.902
Cesarean delivery	1.402 (0.893, 2.201)	0.141	2.105 (0.943, 4.698)	0.069	1.523 (0.700, 3.317)	0.289	1.272 (0.571, 2.841)	0.557
Birth weight	0.999 (0.998, 1.000)	0.036	0.999 (0.997, 1.001)	0.189	0.999 (0.998, 1.001)	0.280	0.999 (0.998, 1.000)	0.188
Assisted reproductive technology	1.721 (0.810, 3.663)	0.158	1.565 (0.332, 7.407)	0.571	4.082 (1.303, 12.821)	0.016	1.438 (0.313, 6.617)	0.641
High IPL III-V (> 25%)								
ID	1.064 (0.742, 1.525)	0.737	1.350 (0.654, 2.786)	0.471	1.018 (0.556, 1.862)	0.955	1.328(0.724, 2.436)	0.359
Cesarean delivery	1.197 (0.876, 1.637)	0.259	1.134(0.659,1.951)	0.650	1.231(0.726, 2.088)	0.441	1.208(0.687, 2.124)	0.511
Birth weight	1.000(0.999, 1.000)	0.205	0.999(0.999, 1.000)	0.264	0.999(0.998, 1.000)	0.258	1.000(0.999, 1.001)	0.675
Assisted reproductive technology	1.129(0.621, 2.053)	0.691	1.437 (0.397, 5.209)	0.581	1.938 (0.759, 4.950)	0.167	1.141(0.398, 3.265)	0.806
High IPL III-V (> 50%)								
ID	1.239 (0.904, 1.698)	0.183	1.202 (0.666, 2.169)	0.541	1.304(0.768, 2.216)	0.326	1.484 (0.855, 2.576)	0.161
Cesarean delivery	1.011 (0.769, 1.328)	0.938	1.164 (0.728, 1.862)	0.525	1.121(0.708, 1.776)	0.626	1.022(0.619, 1.689)	0.932
Birth weight	0.999 (0.999, 1.000)	0.027	0.999(0.999, 1.001)	0.264	0.999(0.998, 1.000)	0.026	1.000(0.999, 1.001)	0.523
Assisted reproductive technology	1.337(0.782, 2.284)	0.288	1.683 (0.594, 4.770)	0.327	1.410(0.558, 3.571)	0.468	2.192(0.852, 5.640)	0.104
High SP/AP ratio (> 10%)								
ID	1.129 (0.660, 1.931)	0.658	1.123 (0.460, 2.742)	0.800	1.403 (0.578, 3.401)	0.454	2.198 (0.722, 6.711)	0.165
Cesarean delivery	1.339 (0.842, 2.132)	0.218	1.414 (0.659, 3.030)	0.374	1.166 (0.516, 2.632)	0.712	1.376 (0.578, 3.279)	0.471
Birth weight	1.000(0.999, 1.000)	0.282	0.999(0.998, 1.001)	0.327	0.998(0.997, 1.000)	0.038	1.001 (0.999, 1.002)	0.208
Assisted reproductive technology	1.664 (0.586, 4.727)	0.339	2.087(0.265, 16.417)	0.484	–	–	1.406 (0.376, 5.263)	0.613
High SP/AP ratio (> 25%)								
ID	1.429 (1.007, 2.029)	0.046	1.385 (0.724, 2.650)	0.325	1.327 (0.721, 2.443)	0.363	1.747 (1.045, 3.078)	0.027
Cesarean delivery	1.064 (0.777, 1.458)	0.699	1.178(0.689,2.012)	0.550	1.315(0.768, 2.254)	0.319	1.280(0.697, 2.353)	0.426
Birth weight	1.000(0.999, 1.000)	0.159	0.999(0.998, 1.000)	0.067	1.000(0.999, 1.001)	0.610	1.000(0.999, 1.001)	0.700
Assisted reproductive technology	1.449 (0.522, 3.673)	0.384	1.518 (0.418, 5.507)	0.526	2.273 (1.258, 4.886)	0.035	1.129(0.356, 3.584)	0.837
High SP/AP ratio (> 50%)								
ID	1.392 (1.013, 1.912)	0.041	1.124 (0.623, 2.026)	0.698	1.068(0.630, 1.810)	0.808	2.133 (1.220, 3.728)	0.008
Cesarean delivery	1.068 (0.810, 1.408)	0.642	1.037 (0.649, 1.659)	0.878	0.999(0.998, 1.000)	0.049	1.214 (0.732, 2.016)	0.452
Birth weight	1.122 (1.025, 1.229)	0.013	0.999(0.998, 1.000)	0.119	1.150(0.725, 1.824)	0.552	1.000(0.999, 1.001)	0.911
Assisted reproductive technology	1.593(0.926, 2.739)	0.093	1.244 (0.449, 3.446)	0.674	1.700(0.683, 4.230)	0.254	1.553 (0.616, 3.912)	0.351

*SES* Socioeconomic status, *OME* Otitismedia with effusion, *ID* Iron deficiency, *AP* Action potential, *SP* Summating potential, *IPL* Interpeak latency

**Table 6:** Multiple linear regression model of iron status (newborn SF and maternal Hb) and SP/AP ratio (wave V) by SES

	Low SES			Moderate SES			High SES		
	*β* (95% CI)	*P*	ad R^2^	*β* (95% CI)	*P*	ad R^2^	*β* (95% CI)	*P*	ad R^2^
For SP/AP ratio (wave V)		< 0.001	0.309		0.031	0.207		< 0.001	0.261
SF	-0.532 (-0.548, -0.516)	< 0.001		-0.442 (-0.459, -0.425)	< 0.001		-0.482 (-0.523, -0.461)	< 0.001	
Hb (g/L)	-0.093 (-0.125, -0.060)	0.086		-0.052 (-0.085, -0.018)	0.352		-0.094 (-0.140, 0.052)	0.126	
Age at delivery (years)	0.037 (-0.046, 0.119)	0.479		-0.061 (-0.135, 0.004)	0.282		0.013 (-0.096, 0.122)	0.829	
Gestational age (wk)	0.015 (-0.180, 0.210)	0.763		0.007 (-0.207, 0.220)	0.900		-0.099 (-0.373, 0.175)	0.075	
Pre-pregnancy BMI (kg/m^2^)	0.024 (-0.293, 0.342)	0.664		-0.050 (-0.369, 0.268)	0.360		-0.149 (-0.501, 0.203)	0.008	
LGFBG (mmol/L) ^*^	-0.013 (-0.610, 0.584)	0.797		-0.034 (-0.695, 0.627)	0.515		0.095 ( -0.890, 0.979)	0.087	
Extra-sport activities during pregnancy	0.009 (-0.829, 0.853)	0.855		-0.144 (-1.986, -0.307)	0.007		-0.009 ( -1.147, 1.129)	0.879	
Smoking before pregnancy	-0.102 (-4.135, 3.931)	0.046		-0.004 (-0.3.184, 4.175)	0.943		0.027 (-8.671, 8.725)	0.627	
Passive smoking during pregnancy	-0.329 (-1.644, 0.194)	0.414		0.074(-0.966, 1.114)	0.156		-0.105 (-1.445, 1.236)	0.068	
Drinking before pregnancy	0.123 (-1.335, 1.581)	0.016		-0.030 (-1.446, 1.386)	0.560		-0.015 (-1.692, 1.663)	0.799	
Mixed diet	0.029 (-0.465, 0.522)	0.557		-0.085 (-0.593, 0.423)	0.102		0.040 ( -0.657, 0.737)	0.469	
Cesarean delivery	-0.038 (-0.886, 0.809)	0.473		0.083 (-0.800, 0.965)	0.144		0.110 (-0.875, 1.195)	0.048	
Assisted reproductive technology	0.045(-1.695, 1.785)	0.370		0.019 (-1.617, 1.655)	0.717		-0.012 (-1.961, 1.937)	0.833	
Hypertension during pregnancy	0.064 (-1.537, 1.664)	0.202		-0.112 (-2.776, 2.551)	0.031		0.030 ( -5.341, 4.401)	0.595	
Birth weight	-0.056 (-0.058, -0.054)	0.301		-0.048(-0.049, -0.046)	0.374		0.039 (0.037, 0.040)	0.486	
Apgar score at 5 min	-0.021 (-2.938, 2.996)	0.668		-0.020 (-2.679, 2.639)	0.712		0.039 (-8.530, 8.607)	0.476	
Female	0.066 (-0.750, 0.881)	0.196		0.134 (-0.687,0.954)	0.010		0.003 (-1.097, 1.303)	0.956	

^*^ LG: logarithm transformation, *SES* Socioeconomic status, *FBG *Fasting blood glucose, *BMI *Body mass index, *Hb *Hemoglobin, *SF *Serum ferritin

Table 7 showed the multiple logistic regression of relationship between children OME after COVID-19 as dependent variable and ID as independent variables after controlling for Birth weight, cesarean delivery and assisted reproductive technology. The risk of OME in children with positive COVID-19 was significantly higher in those with negative COVID-19 (*P* < 0.05). Among three SES, COVID-19 exhibited most statistical association with the risk of OME in high SES [OR (95% CI): 6.751 (1.876, 14.288)]. The risk of OME in children who were ID in utero was significantly higher those in NIS (*P* < 0.05). Among three SES, ID exhibited lowest statistical association with the risk of OME after COVID-19 in low SES [OR (95% CI): 6.622 (1.567, 13.778)]. But, no statistical association between ID and the risk of OME before COVID-19 was found in all SES.

**Table 7:** Associations between ID in different SES and children OME in a multiple logistic regression by SES

Multivariate regression	All Low SES				Moderate SES		High SES	
	OR (95% CI)	*P*	OR (95% CI)	*P*	OR (95% CI)	*P*	OR (95% CI)	*P*
For OME after COVID-19								
COVID-19	6.634(1.891, 23.279)	0.003	15.588(3.695, 35.767)	0.001	18.098(3.837, 35.355)	0.000	6.751 (1.876, 14.288)	0.003
ID	3.300 (1.379, 7.874)	0.007	6.622 (1.567, 13.778)	0.010	2.577(0.929, 7.143)	0.069	2.874 (1.085, 7.634)	0.034
Cesarean delivery	1.266 (0.725, 2.212)	0.407	1.567(0.762, 3.236)	0.222	1.212(0.434, 3.390)	0.713	1.036 (0.582, 2.033)	0.918
Birth weight	0.999(0.998, 1.000)	0.244	0.999(0.998, 1.000)	0.216	0.999(0.997, 1.001)	0.490	1.000 (0.998, 1.001)	0.544
Assisted reproductive technology	1.016(0.346, 4.287)	0.977	2.957(0.362, 4.171)	0.312	-	-	1.425 (0.460, 4.405)	0.540
For OME before COVID-19								
ID	1.160 (0.372, 3.610)	0.798	3.861 (0.460, 32.258)	0.213	2.418 (0.627,9.324)	0.200	1.488 (0.406, 5.464)	0.547
Cesarean delivery	1.316 (0.480, 3.610)	0.595	1.058(0.313, 3.584)	0.927	1.201 (0.285, 5.062)	0.803	1.488(0.486, 4.566)	0.486
Birth weight	0.999(0.997, 1.001)	0.252	1.000(0.997, 1.002)	0.716	0.999(0.997, 1.002)	0.621	0.999 (0.997, 1.001)	0.243
Assisted reproductive technology	1.088 (0.140, 8.456)	0.936	1.332(0.151, 11.745)	0.751	0.450 (0.051, 3.941)	0.471	-	-

Some data were not shown because the number was small

*SES* Socioeconomic status, *OME* Otitismedia with effusion, *COVID-19* Corona Virus Disease 2019, *ID* Iron deficiency

Table 8 showed the multiple logistic regression of relationship between possible HHL criteria in newborn as dependent variable and ID as independent variables after controlling for Birth weight, cesarean delivery and assisted reproductive technology. The risk of high absolute latency wave V (> 50%) in ID newborn was significantly higher in those with NIS in low [OR (95% CI): 2.408 (1.295, 4.476)] and high SES [OR (95% CI): 1.984 (1.117, 3.524)] (*P* < 0.05). The risk of high absolute latency wave V (> 50%) in newborns born by caesarean section was significantly higher those born naturally in moderate SES (*P* < 0.05). The risk of high SP/AP ratio (> 25%) and (> 50%) in ID newborns was significantly higher in those with NIS in high SES [OR (95% CI): 1.747 (1.045, 3.078)] and [OR (95% CI): 2.133 (1.220, 3.728)] (*P* < 0.05). The risk of high IPL III-V (> 10%), high SP/AP ratio (> 25%) in newborns born by assisted reproduction was significantly higher those by natural conception in moderate SES (*P* < 0.05).

**Table 8:** Associations between ID in different SES and newborn HHL [High absolute latency wave V, IPL III-V, and SP/AP ratio] in a multiple logistic regression by SES

Multivariate regression	All		Low SES		Moderate SES		High SES	
	OR (95% CI)	*P*	OR (95% CI)	*P*	OR (95% CI)	*P*	OR (95% CI)	*P*
For high absolute latency wave V (> 10%)								
ID	1.214 (1.379, 0.702)	0.489	1.263 (0.481, 3.319)	0.636	1.580 (0.617, 4.049)	0.340	1.357 (0.515, 3.571)	0.536
Cesarean delivery	1.130 (0.721, 1.771)	0.594	2.153 (0.981, 4.723)	0.056	1.393 (0.651, 2.976)	0.393	1.002 (0.433, 2.319)	0.997
Birth weight	1.000 (1.000, 1.001)	0.331	1.001 (0.999, 1.002)	0.314	1.000 (0.998, 1.001)	0.490	1.001 (1.000, 1.003)	0.118
Assisted reproductive technology	2.080 (0.635, 6.809)	0.226	–-	–-	1.078 (0.234, 4.972)	0.923	2.308 (0.292, 18.230)	0.428
High absolute latency wave V (> 25%)								
ID	1.204 (0.844, 1.718)	0.305	1.886 (0.994, 3.581)	0.052	1.164 (0.633, 2.141)	0.626	1.160 (0.617, 2.183)	0645
Cesarean delivery	1.020 (0.755, 1.400)	0.899	1.561 (0.909, 2.692	0.106	1.383 (0.816, 2.342)	0.228	1.115 (0.618, 2.012)	0.717
Birth weight	1.000 (0.999, 1.000)	0.704	1.001 (1.000, 1.002)	0.090	0.999 (0.998, 1.000)	0.018	1.000 (0.999, 1.001)	0.699
Assisted reproductive technology	1.949 (0.904, 4.042)	0.073	2.199 (0.478, 10.111)	0.311	1.259 (0.435, 3.64)	0.671	3.216(0.723, 14.302)	0.125
High absolute latency wave V (> 50%)								
ID	1.680 (1.220, 2.313)	0.001	2.408 (1.295, 4.476)	0.005	1.293 (0.757, 2.207)	0.347	1.984 (1.117, 3.524)	0.019
Cesarean delivery	1.164 (0.883, 1.400)	0.286	1.213 (0.754, 1.951)	0.425	1.700 (1.070, 2.700)	0.025	1.067 (0.645, 1.767)	0.799
Birth weight	1.000 (0.999, 1.000)	0.254	1.000 (0.999, 1.001)	0.914	0.999 (0.998, 1.000)	0.047	1.000 (0.999, 1.001)	0.945
Assisted reproductive technology	1.949 (0.904, 4.042)	0.073	2.184 (0.726, 6.566)	0.164	1.220 (0.487, 3.056)	0.672	2.358 (0.881, 6.309)	0.088
For high IPL III-V (> 10%)								
ID	1.285 (0.735, 2.247)	0.379	1.012 (0.360, 2.840)	0.982	2.717 (0.882, 8.403)	0.082	1.056 (0.442, 2.525)	0.902
Cesarean delivery	1.402 (0.893, 2.201)	0.141	2.105 (0.943, 4.698)	0.069	1.523 (0.700, 3.317)	0.289	1.272 (0.571, 2.841)	0.557
Birth weight	0.999 (0.998, 1.000)	0.036	0.999 (0.997, 1.001)	0.189	0.999 (0.998, 1.001)	0.280	0.999 (0.998, 1.000)	0.188
Assisted reproductive technology	1.721 (0.810, 3.663)	0.158	1.565 (0.332, 7.407)	0.571	4.082 (1.303, 12.821)	0.016	1.438 (0.313, 6.617)	0.641
High IPL III-V (> 25%)								
ID	1.064 (0.742, 1.525)	0.737	1.350 (0.654, 2.786)	0.471	1.018 (0.556, 1.862)	0.955	1.328(0.724, 2.436)	0.359
Cesarean delivery	1.197 (0.876, 1.637)	0.259	1.134(0.659,1.951)	0.650	1.231(0.726, 2.088)	0.441	1.208(0.687, 2.124)	0.511
Birth weight	1.000(0.999, 1.000)	0.205	0.999(0.999, 1.000)	0.264	0.999(0.998, 1.000)	0.258	1.000(0.999, 1.001)	0.675
Assisted reproductive technology	1.129(0.621, 2.053)	0.691	1.437 (0.397, 5.209)	0.581	1.938 (0.759, 4.950)	0.167	1.141(0.398, 3.265)	0.806
High IPL III-V (> 50%)								
ID	1.239 (0.904, 1.698)	0.183	1.202 (0.666, 2.169)	0.541	1.304(0.768, 2.216)	0.326	1.484 (0.855, 2.576)	0.161
Cesarean delivery	1.011 (0.769, 1.328)	0.938	1.164 (0.728, 1.862)	0.525	1.121(0.708, 1.776)	0.626	1.022(0.619, 1.689)	0.932
Birth weight	0.999 (0.999, 1.000)	0.027	0.999(0.999, 1.001)	0.264	0.999(0.998, 1.000)	0.026	1.000(0.999, 1.001)	0.523
Assisted reproductive technology	1.337(0.782, 2.284)	0.288	1.683 (0.594, 4.770)	0.327	1.410(0.558, 3.571)	0.468	2.192(0.852, 5.640)	0.104
High SP/AP ratio (> 10%)								
ID	1.129 (0.660, 1.931)	0.658	1.123 (0.460, 2.742)	0.800	1.403 (0.578, 3.401)	0.454	2.198 (0.722, 6.711)	0.165
Cesarean delivery	1.339 (0.842, 2.132)	0.218	1.414 (0.659, 3.030)	0.374	1.166 (0.516, 2.632)	0.712	1.376 (0.578, 3.279)	0.471
Birth weight	1.000(0.999, 1.000)	0.282	0.999(0.998, 1.001)	0.327	0.998(0.997, 1.000)	0.038	1.001 (0.999, 1.002)	0.208
Assisted reproductive technology	1.664 (0.586, 4.727)	0.339	2.087(0.265, 16.417)	0.484	–	–	1.406 (0.376, 5.263)	0.613
High SP/AP ratio (> 25%)								
ID	1.429 (1.007, 2.029)	0.046	1.385 (0.724, 2.650)	0.325	1.327 (0.721, 2.443)	0.363	1.747 (1.045, 3.078)	0.027
Cesarean delivery	1.064 (0.777, 1.458)	0.699	1.178(0.689,2.012)	0.550	1.315(0.768, 2.254)	0.319	1.280(0.697, 2.353)	0.426
Birth weight	1.000(0.999, 1.000)	0.159	0.999(0.998, 1.000)	0.067	1.000(0.999, 1.001)	0.610	1.000(0.999, 1.001)	0.700
Assisted reproductive technology	1.449 (0.522, 3.673)	0.384	1.518 (0.418, 5.507)	0.526	2.273 (1.258, 4.886)	0.035	1.129(0.356, 3.584)	0.837
High SP/AP ratio (> 50%)								
ID	1.392 (1.013, 1.912)	0.041	1.124 (0.623, 2.026)	0.698	1.068(0.630, 1.810)	0.808	2.133 (1.220, 3.728)	0.008
Cesarean delivery	1.068 (0.810, 1.408)	0.642	1.037 (0.649, 1.659)	0.878	0.999(0.998, 1.000)	0.049	1.214 (0.732, 2.016)	0.452
Birth weight	1.122 (1.025, 1.229)	0.013	0.999(0.998, 1.000)	0.119	1.150(0.725, 1.824)	0.552	1.000(0.999, 1.001)	0.911
Assisted reproductive technology	1.593(0.926, 2.739)	0.093	1.244 (0.449, 3.446)	0.674	1.700(0.683, 4.230)	0.254	1.553 (0.616, 3.912)	0.351

*SES* Socioeconomic status, *OME* Otitismedia with effusion, *ID* Iron deficiency, *AP* Action potential, *SP* Summating potential, *IPL* Interpeak latency

The ID prevalence (81.5%) of mother-newborns in low SES was the highest among three SES (Supplementary Table 2). Supplementary Table 10 showed the mediation analyses of the associations between SES and auditory neural maturation by iron status. But iron status did not play a mediation role in the association between SES and auditory neural maturation as well as related parameters. The proportion of the mediation roles of ID over the total effect of low SES on HHL was 21.82% (wave V), 10.67% (IPL III-V) and 19.23% (SP/AP ratio), respectively.

Supplementary Table 11 showed combined effects of SES and iron status on risk of children OME or neonatal HHL as well as auditory neural maturation related parameters. The reference was composed of IS mother-newborns with high SES. The risk of children OME after COVID-19 was the highest among ID mother-newborns with low SES [OR (95% CI): 1.624 (1.133, 3.667)]. Then, for high absolute latency wave V (one of the HHL-related parameters), the risk of HHL in ID mother-newborns regardless of SES was higher than those who were in IS, moreover, the highest risk was in ID mother-newborns with low SES [OR (95% CI): 2.448 (1.304, 4.598)]. For high SP/AP ratio (wave V) (one of the HHL-related parameters), the risk of HHL in ID mother-newborns with low or high SES was higher than those who were in IS or ID with moderate SES. Specially, the highest risk was in ID mother-newborns with high SES [OR (95% CI): 2.184 (1.254, 3.805)]. In addition, similar significant combined effects were observed between ID and SES on absolute latency wave V and SP/AP ratio.

Supplementary Tables 12 and 13 showed the results of sensitivity analyses. After excluding children with adenoid hypertrophy, chronic sinusitis, and allergic rhinitis, the associations of ID with OME prevalence did not change substantially. But the effect estimate of SES on OME risk was no longer statistically significant (Supplementary Table 12). No substantial changes were found between ID and OME prevalence by additionally adjusting for more COVID-19 Symptoms, regardless of SES tertiles (Supplementary Table 13).

**Fig. 1:**
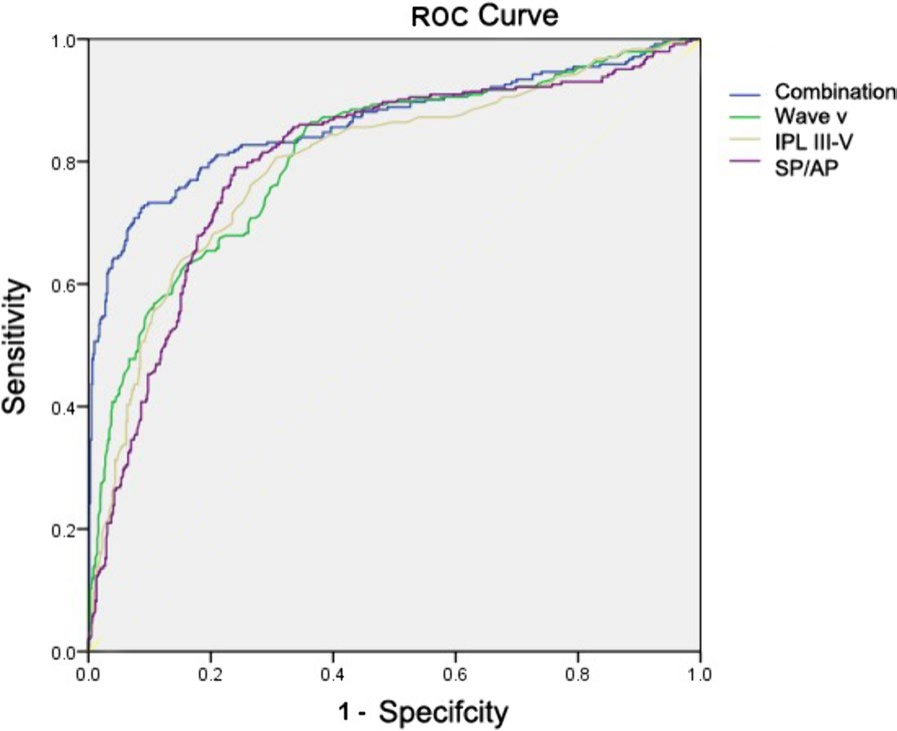
ROC curves of absolute latency wave V, IPL III-V, and SP/AP ratio for the diagnosis of neonatal HHL. ROC: receiver operating characteristic, IPL: interpeak latency, HHL: hidden hearing loss

Here, mother-newborn exposed to ID were considered as potential HHL patients. ROC curve and the area under the ROC curve (AUC) were calculated by forward LR method (Fig. 1). According to the previous analysis of predictive factors in this study, absolute latency wave V, IPL III-V, and SP/AP ratio may be potential standard indicators to diagnose HHL. The AUC values of absolute latency wave V, IPL III-V and SP/AP ratio (wave V) were 0.811 (95%CI: 0.777–0.845), 0.798 (95%CI: 0.763–0.833), and 0.801 (95%CI: 0.767–0.836), respectively (*p* < 0.001). The cut off values of absolute latency wave V, IPL III-V and SP/AP ratio were 6.39 ms, 2.33 ms and 27.20%. The Youden indices of the above indicators were 0.473, 0.487, and 0.471. The AUC combination value of the above indicators was 0.861 (95% CI 0.829–0.894), which was superior to that of the other single indicators in predicting HHL. The combination indicators had higher diagnostic and predictive values for neonatal HHL. The sensitivity was 72.40% and the specifcity was 91.40%.

## Discussion

As expected, ID delayed auditory neural maturation, which was manifested by absolute latency wave V, IPL III-V, and SP/AP ratio (wave V), which were combined as HHL criterion. To our knowledge, this study was the first to prove that low SES strengthened the adverse effect of ID on auditory nerve maturation and related ABR and ECochG parameters, estimate the joint associations of ID and low SES with the highest risk of HHL and the highest levels of HHL-related parameters in newborns. Moreover, after COVID-19 were positive, preschool children who experience ID in neonatal period were more likely to suffer from OME. Unexpectedly, high SES also showed similar risk effects.

From the above two easily neglected public health problems, this study attempted to find the associations of two easily neglected public health problems (ID and HHL) in the process of auditory nerve maturation with social factors (SES), more importantly, to estimate the diagnostic criteria of HHL. Based on the previous criteria for neonatal ID [9, 10, 20], we combined with maternal ID criteria, and presented ID from the third trimester to birth by combination of maternal Hb and neonatal SF (105 g/L ≤ maternal Hb < 120 g/L, neonatal SF ≤ 75 ng/mL). However, the role of neonatal SF was higher than that of maternal Hb in HHL risk. Nearly a quarter of newborns showed ID, which was similar with the observations in previous studies (19.4%-30.3%). [9, 10, 20]. Except that the levels of absolute latency wave V, IPL III-V, and SP/AP ratio of ID were higher than those of NIS, ID mother-newborns had a higher proportion of low SES. Unexpectedly, compared with high SES, a higher proportion of moderate SES participated in this study. It may be that high SES in China pays more attention to their family information today, even if we protected the privacy of respondents and facilitated the anonymous analysis of the data. Then, both of neonatal SF and maternal Hb in moderate SES were the highest among SES tertiles. Unexpectedly, SF and Hb levels in high SES were the lowest among tertiles SES. ICC analysis indirectly reflected that the variability of SF among tertiles SES was higher than that of Hb. A special phenomenon has emerged in contemporary China. That is, high SES faced the most mental stress from work or family based on our previous study [21]. Moreover, high SES had the highest proportion smoking and drinking, which might indirectly reflect that high SES had more mental stress. The psychological stress may be one of the factors that increases sensorineural, sudden hearing loss, and tinnitus, vertigo [22]. Because we found the ID group had a higher proportion of high SES and lower proportion of moderate SES than NIS group, the independent variables of we were interested in included iron status and SES indicators. The covariates included hypertension, BMI, activity, smoking, and drinking. These covariates were selected because hypertension, BMI, activity, smoking, and drinking have been shown to be significantly associated with hearing loss [23–26].

The reason why we took COVID-19 positive as covariant was that we wanted to take COVID-19 as a short-term exposure risk factor. The Chinese government closed and unsealed the COVID-19 on December 14, 2022. As a result, almost all Chinese people were positive in the same period (from December 2022 to March 2023), and this phenomenon was unique in the world. The incidence of OME in the outpatient of our hospital was significantly higher than that in other years in the same period. This verifies that if developmental ID may cause newborn HHL (from November 2013 to May 2017), it is more likely to lead to hearing loss when encountering certain exposure stimuli during later growth.

The main contribution of this study was to initially find the indicators and standard values for diagnosis of HHL in newborns. Despite the above values were obtained in ID status, the ID status was closer to the natural status, which was subclinical status after all. Different from previous “the standard of HHL Diagnosis” literatures after high noise exposure [16, 17, 27, 28], they presented HHL before showing an elevated threshold at high frequencies by using thresholds in noise or combined diagnostic indicators in adults. ABR and ECochG were also used to reflect auditory neural maturation of newborns in this study, which showed that absolute latency wave V, IPL III-V, and SP/AP ratio may be useful in the diagnosis of HHL. These results were partly similar with those of other studies, but also different. Two prospective cohort studies in infants born at ≥ 34 or 35 weeks of gestation in India reinforced the likely importance of iron status as determinants of hearing at the brainstem level. In utero ID infants ≥ 34 weeks of gestation prolonged wave V latencies and IPL III–V and I–V [10], but ID infants ≥ 35 weeks of gestation did not express significant differences in wave or interpeak latencies compared with NIS at birth or at 4–6 months of age [29]. Not only our results found that ID prolonged wave V latencies and IPL III–V, but also increased SP/AP ratio. These differences in the brainstem results may be due to differences in population characteristics [29].

Our study did not include distortion-product otoacoustic emissions (DPOAE) and categorical loudness scaling (CLS). On the one hand, the simpler measurements were more beneficial for subjects to accept participation, on the other hand, DPOAE mainly reflects the function of outer hair cells (OHCs), and ECochG is more sensitive than DPOAE. CLS belongs to physical measures, reflects both OHC and IHC integrity [17]. Excluding middle ear lesions, the abnormal results of ABR and ECochG reflect the pathological changes of auditory pathways except OHCs. Among them, wave I, III, and V reflects auditory nerve activity, hypothalamus, and auditory brainstem, respectively [28]. In addition, the newborns with negative initial hearing screening are at high risk of undergoing hearing tests. To minimize the risk, this study transported the newborn from the delivery room to the soundproof room, which was completely enclosed and accompanied by parents and doctors, and arrived within 5 min. The stimulation electrode was placed under the supervision of experienced otolaryngologists and obstetricians. On the one hand, we reduced the stimulation sound pressure level, and on the other hand, we required ECochG completion within 10 min.

The absolute latency of wave I is suitable for diagnosis of HHL in population, but it is not suitable for diagnosis in individuals. The absolute latency of wave V is used as a marker of cochlear synaptopathy. IPL I–III, III–V and I–V reflects myelination, synaptic maturation, and whole brainstem, respectively. The hair cells generated SP, which reflects both OHC and IHC integrity. The cochlear neurons generated AP, which reflects ANFs integrity. So, SP/AP ratio, which reflects hair cells integrity relative to the ANFs integrity, which has previously been used as diagnostic value (> 40%) in Ménière’s disease, which has also used in inferring cochlear synaptopathy and HHL (> 29%) [30], which was similar with our data of SP/AP ratio (> 27%). The data from this study confirmed that ID during pregnancy may be useful for the prediction of HHL in newborns, that is, by increased wave V latencies, IPL III–V, and SP/AP ratio, reflecting cochlear synaptopathy. Considering that volatility of these three indicators (e.g., differences in head size, electrode contact), the combined these indictors may be superior to predict HHL by the AUC combination value.

SES determines their access to the diversity of live choices and medical resources. Because of the moderate correlation between SES and SF, as well as the strong independent correlation between SES and HHL risk, we explored the highest combined effects of ID mother-newborns with low SES on newborns HHL risk and HHL-related parameters.

This study had several limitations. First, the enrolled pregnant women in the cohort study were from a single tertiary care centre from Liaoning Province in Northeast China, which belonged to the heavy industrial and economically backward areas with severe pollution. The results might not reflect the situation of other regions in China.Second, no specific component of SES was analyzed to significantly strengthen the risk of ID on neonatal HHL. All enrolled newborns were from two-parent families, we did not choose newborns from single-parent households. However, single-parent households might enhance newborns hearing loss. Third, this study did not report residential exposure to traffic noise during pregnancy, which might affect children health including hearing [31].

## Conclusion

Overall, iron status and SES were independently associated with auditory neural maturation and the prediction of infant HHL in Northeast of China. The low and high SES strengthened the detrimental effect of ID, on delaying auditory neural maturation and the infant HHL risk, by HHL-related parameters (the higher levels of absolute latency of wave V, IPL III–V, and SP/AP ratio), especially for low SES and ID. These may provide a perspective for the prediction of HHL in newborns.

### Supplementary Information

**Supplementary Material 1.**

## Data Availability

The datasets used and/or analyzed during this study are available from the corresponding author on reasonable request.

## Abbreviations

ABR: Auditory brainstem response testing
ANOVA: Analysis of variance
ANF: Auditory nerve fibers
AP: Action potentials
BMI: Body mass index
COVID-19: Corona Virus Disease 2019
CI: Confidence interval
CLS: Categorical loudness scaling
DPOAE: Distortion-product otoacoustic emissions
ECochG: Electrocochleography
FBG: Fasting blood glucose
Hb: Hemoglobin
HHL: Hidden hearing loss
IHC: Inner hair cells
IPL: Interpeak latency
ID: Iron deficiency
IDA: Iron deficiency anemia
NIS: Normal iron status
SES: Socioeconomic status
SF: Serum ferritin
SP: Summting potentials
SD: Standard deviations
OR: Odds ratio
OHC: Outer hair cells
OME: Otitis media with effusion
