# Supplementary material for: Iron deficiency at birth and risk of hidden hearing loss in infants modification by socioeconomic status: mother-newborn cohort in Shenyang, China
Source: BMC Public Health. 2024 Apr 3;24:953. doi: 10.1186/s12889-024-18439-4 (PMC10988964; doi:10.1186/s12889-024-18439-4)
Supplement: Supplementary file 1 — Supplementary Material 1. [file 12889_2024_18439_MOESM1_ESM.docx]

Supplementary Table 1 **Baseline characteristics of mother-newborn-child** **by SES tertiles**

|  | Low SES 298 | Moderate SES 304 | High SES 259 | *p*-Value |
| --- | --- | --- | --- | --- |
| SF | 87.42 ± 26.21 | 93.82 ± 24.40 ^*^ | 86.04 ± 25.19 | <0.001 |
| Hb | 123.19 ± 12.95 | 124.44 ± 12.93 | 118.91 ± 12.84 ^*^ | <0.001 |
| BMI | 23.49 ± 1.57 | 22.81 ± 1.33 ^*^ | 22.29 ± 1.54 ^#^ | <0.001 |
| Birth weight | 3542.65 ± 244.61 | 3451.64 ± 266.52 ^*^ | 3480.35 ± 302.89 | <0.001 |
| Age at delivery (years) | 28.82 ± 5.22 | 29.09 ± 5.09 | 29.65 ± 5.06 | 0.140 |
| Gestational age (wk) | 39.30 ± 2.01 | 39.41 ± 1.96 | 39.47 ± 1.96 | 0.604 |
| ID | 243(81.5) | 226 (74.3) | 184 (71.6) | 0.016 |
| Male sex | 181 (60.7) | 186 (61.2) | 157 (61.1) | 0.993 |
| Cesarean delivery | 182 (61.1) | 169 (55.6) | 147(57.2) | 0.378 |
| Assisted reproductive technology | 16(5.4) | 21(6.9) | 22(8.6) | 0.333 |
| OME |  |  |  | 0.576 |
| No | 241(91.3) | 238(90.2) | 216(88.9) |  |
| After COVID-19 | 20(7.6) | 18(6.8) | 21(8.6) |  |
| Before COVID-19 | 3(1.1) | 8(3.0) | 6(2.5) |  |
| COVID | 266(99.6) | 256(96.2) | 241(98.8) | 0.01 |

Data are shown as n (%) or mean ± SD. *^, #^ Means with different symbol are significant (*P* < 0.001). OME: otitismedia with effusion, COVID-19: Corona Virus Disease 2019, ID: iron deficiency, FBG: fasting blood glucose, BMI: body mass index, Hb: hemoglobin, SF: serum ferritin, SES: socioeconomic status.

**Supplementary Table 2 Distributions of neonatal SF and maternal Hb according to SES.**

| SF (ng/mL) | Mean ± SD |  | Pearson correlation |  | ICC (95%CI) |
| --- | --- | --- | --- | --- | --- |
|  |  | Low | Moderate | High |  |
| All SES (*n* = 859) | 89.27 ± 25.47 |  |  |  | 0.58 (0.51-0.64) |
| Low SES (*n* = 298) | 87.42 ± 26.21 | 1 | 0.37 | 0.46 |  |
| Moderate SES (*n* = 304) | 93.82 ± 24.40 |  | 1 | 0.26 |  |
| High SES (*n* = 257) | 86.04 ± 25.19 |  |  | 1 |  |
| *P* | < 0.001 |  |  |  |  |
| Hb (g/L) |  |  |  |  |  |
| All SES (*n* = 859) | 122.36 ± 13.10 |  |  |  | 0.38 (0.30-0.46) |
| Low SES (*n* = 298) | 123.19 ± 12.95 | 1 | 0.63 | 0.66 |  |
| Moderate SES (*n* = 304) | 124.44 ± 12.93 |  | 1 | 0.43 |  |
| High SES (*n* = 257) | 118.91 ± 12.84 |  |  | 1 |  |
| *P* | < 0.001 |  |  |  |  |

All the correlations were statistically significant (*P*<0.001). ICC: intraclass correlation coefficient, Hb: hemoglobin, SF: serum ferritin, SES: socioeconomic status.

**Supplementary Table3 Correlations between iron status (neonatal SF, maternal Hb) and auditory neural maturation (****wave** V**, IPL** III-V**, and SP/AP ratio) by SES.**

|  | All (*n* = 859) | | Low SES (*n* = 298) | | Moderate SES (*n* = 304) | | High SES (*n* = 257) | |
| --- | --- | --- | --- | --- | --- | --- | --- | --- |
|  | SF | Hb | SF | Hb | SF | Hb | SF | Hb |
| Absolute latency wave V (ms) ^Δ^ | -0.423 | -0.267 | -0.395 | -0.300 | -0.398 | -0.222 | -0.471 | -0.272 |
| IPL III-V (ms) ^#^ | -0.442 | -0.256 | -0.447 | -0.310 | -0.387 | -0.195 | -0.482 | -0.235 |
| SP/AP ratio (wave V) ^Δ^ | -0.487 | -0.237 | -0.555 | -0.262 | -0.464 | -0.172 | -0.497 | -0.263 |

^*^ *P* < 0.01 ^#^ Spearman correlation coefficient ^Δ^ Pearson correlation coefficient Hb: hemoglobin, SF: serum ferritin, SES: socioeconomic status, AP: action potential, SP: summating potential, IPL: interpeak latency.

**Supplementary Table 4 Comparisons of absolute latency wave** V **by the quartile of SF and Hb concentrations.**

| Iron status quartile  (range) | Absolute latency wave V (ms) | | | |
| --- | --- | --- | --- | --- |
|  | All | Low SES | Moderate SES | High SES |
|  | *n* mean ± SD | *n* mean ± SD | *n* mean ± SD | *n* mean ± SD |
| All | 859 5.95 ± 0.82 | 298 5.97 ± 0.83 | 304 5.88 ± 0.78 | 257 6.00 ± 0.84 |
| SF (ng/mL) |  |  |  |  |
| Q1:22-74 | 216 6.55 ± 0.75 | 90 6.47 ± 0.81 | 51 6.64 ± 0.65 | 75 6.58 ± 0.74 |
| Q2:74-89 | 211 5.83 ± 0.79 | 69 5.95 ± 0.75 | 73 5.77 ± 0.82 | 69 5.78 ± 0.80 |
| Q3:90-103 | 216 5.73 ± 0.61 | 69 5.59 ± 0.66 | 89 5.77 ± 0.55 | 58 5.84 ± 0.60 |
| Q4:104-208 | 216 5.68 ± 0.79 | 70 5.72 ± 0.78 | 91 5.66 ± 0.77 | 55 5.66 ± 0.86 |
| *P*^Δ^ | 0.001 | 0.001 | 0.001 | 0.001 |
| Hb (g/L) |  |  |  |  |
| Q1:93-112 | 216 6.25 ± 0.84 | 68 6.29 ± 0.92 | 61 6.15 ± 0.77 | 87 6.28 ± 0.83 |
| Q2:113-122 | 226 6.01 ± 0.79 | 75 6.13 ± 0.75 | 76 5.80 ± 0.80 | 75 5.94 ± 0.83 |
| Q3: 123-132 | 227 5.79 ± 0.74 | 86 5.81 ± 0.72 | 87 5.71 ± 0.71 | 54 5.87 ± 0.81 |
| Q4:133-157 | 190 5.73 ± 0.80 | 69 5.70 ± 0.82 | 80 5.78 ± 0.79 | 41 5.70 ± 0.80 |
| *P*^Δ^ | < 0.05 | < 0.05 | < 0.05 | < 0.05 |

^Δ^ *P*-value were calculated by Scheffe test of ANOVA. Hb: hemoglobin, SF: serum ferritin, SES: socioeconomic status.

**Supplementary Table 5 Comparisons of IPL III-V by the quartile of SF and Hb concentrations.**

|  | IPL III-V (ms) | | | |
| --- | --- | --- | --- | --- |
|  | All | Low SES | Moderate SES | High SES |
| Iron status quartile  (range) | *n* Median (*P*_25_-*P*_75_) | *n* Median (*P*_25_-*P*_75_) | *n* Median (*P*_25_-*P*_75_) | *n* Median (*P*_25_-*P*_75_) |
| All | 859 2.09 (1.89-2.37) | 298 2.27(1.91-2.43) | 304 2.18 (1.90-2.31) | 257 2.09(1.88-2.46) |
| SF (ng/mL) |  |  |  |  |
| Q1:22-74 | 216 2.63 (2.06-3.06) | 90 2.53(2.02-3.07) | 51 2.61(2.15-3.16) | 75 2.66(2.06-3.05) |
| Q2:74-89 | 211 2.08 (1.88-2.33) | 69 2.09(1.88-2.34) | 73 2.08(1.93-2.33) | 69 2.06 (1.88-2.33) |
| Q3:90-103 | 216 2.06 (190-2.34) | 69 2.08(1.93-2.24) | 89 2.03 (1.88-2.16) | 58 2.07(1.83-2.30) |
| Q4:104-208 | 216 1.97 (1.83-2.17) | 70 1.97(1.83-2.16) | 91 1.82(2.01-2.19) | 55 1.96(1.83-2.15) |
| *P*^Δ^ | 0.001 | 0.001 | 0.001 | 0.001 |
| Hb (g/L) |  |  |  |  |
| Q1:93-112 | 216 2.25 (2.00-2.96) | 68 2.33(2.02-3.11) | 61 2.14(2.01-2.46) | 87 2.26(1.98-2.77) |
| Q2:113-122 | 226 2.13 (1.91-2.46) | 75 2.12(1.92-2.53) | 76 2.14(1.91-2.36) | 75 2.13 (1.87-2.69) |
| Q3: 123-132 | 227 2.05 (1.87-2.29) | 86 2.08(1.86-2.28) | 87 2.02 (1.87-2.24) | 54 2.06(1.81-2.35) |
| Q4:133-157 | 190 2.02 (1.84-2.21) | 69 1.99(1.85-2.20) | 80 2.02(1.83-2.21) | 41 2.02(1.85-2.23) |
| *P*^Δ^ | 0.001 | 0.001 | 0.011 | 0.007 |

^#^ *P*-value were calculated by Kruskal-Wallis H test. Hb: hemoglobin, SF: serum ferritin, SES: socioeconomic status, IPL: interpeak latency.

**Supplementary Table 6 Comparisons of SP/AP ratio (wave** V**) by the quartile of SF and Hb concentrations.**

| Iron status quartile  (range) | SP/AP ratio (wave V) (ms) | | | | | | |  |
| --- | --- | --- | --- | --- | --- | --- | --- | --- |
|  | All | Low SES | | Moderate SES | | High SES | |  |
|  | *n* mean ± SD | | *n* mean ± SD | | *n* mean ± SD | | *n* mean ± SD | |
| All | 859 27.75 ± 4.28 | 298 27.76 ± 4.00 | | 304 27.56 ±3.96 | | 257 27.97 ±4.92 | |  |
| SF (ng/mL) |  |  | |  | |  | |  |
| Q1:22-74 | 216 31.55 ± 4.08 | 90 31.15 ± 3.22 | | 51 31.40 ±3.76 | | 75 32.13 ± 5.09 | |  |
| Q2:74-89 | 211 27.62 ± 3.53 | 69 27.95 ± 3.47 | | 73 27.41 ± 3.21 | | 69 27.52 ± 3.93 | |  |
| Q3:90-103 | 216 26.19 ± 3.68 | 69 25.67 ± 2.85 | | 89 27.13 ± 4.04 | | 58 25.37 ± 3.70 | |  |
| Q4:104-208 | 216 25.64 ± 3.05 | 70 25.26 ± 3.16 | | 91 25.96 ± 3.08 | | 55 25.58 ± 2.86 | |  |
| *P*^Δ^ | 0.001 | 0.001 | | 0.001 | | 0.001 | |  |
| Hb (g/L) | 859 27.75 ± 4.28 | 298 27.76 ± 4.00 | | 304 27.56 ± 3.96 | | 257 27.97 ± 4.92 | |  |
| Q1:93-112 | 216 29.27 ± 4.63 | 68 29.14 ± 4.13 | | 61 29.23 ± 4.23 | | 87 29.90 ± 5.27 | |  |
| Q2:113-122 | 226 28.29 ± 4.48 | 75 28.58 ± 3.82 | | 76 27.68 ± 3.86 | | 75 28.60 ± 5.53 | |  |
| Q3:123-132 | 227 26.55 ± 3.57 | 86 26.84 ± 3.84 | | 87 26.42 ± 3.29 | | 54 26.30 ± 3.61 | |  |
| Q4:133-157 | 190 26.82 ± 3.76 | 69 26.64 ± 3.72 | | 80 27.42 ± 3.96 | | 41 25.95 ± 2.88 | |  |
| *P*^Δ^ | < 0.05 | < 0.05 | | < 0.05 | | < 0.05 | |  |

^Δ^ *P*-value were calculated by Scheffe test of ANOVA. Hb: hemoglobin, SF: serum ferritin, SES: socioeconomic status.

**Supplementary Table 7 Associations between neonatal SF, maternal Hb and neonatal absolute latency wave** V**, IPL** III-V**, and SP/AP ratio (wave** V**) in low SES.**

| Iron status | Low SES  *n* | Absolute latency wave V |  | IPL III-V |  | SP/AP ratio |  |
| --- | --- | --- | --- | --- | --- | --- | --- |
|  |  | *β* (95% CI) | *P* | *β* (95% CI) | *P* | *β* (95% CI) | *P* |
| SF (ng/mL) |  |  |  |  |  |  |  |
| Q1 | 90 | Ref. | <0.001 | Ref. | <0.001 | Ref. | <0.001 |
| Q2 | 69 | -0.518 (-0.756, -0.280) |  | -0.064 (-0.090, -0.037) |  | -3.196 (-4.200, -2.193) |  |
| Q3 | 69 | -0.875 (-1.113, -0.637) |  | -0.082 (-0.108, -0.056) |  | -5.476 (-6.479, -4.473) |  |
| Q4 | 70 | -0.747 (-0.984, -0.510) |  | -0.104 (-0.130, -0.078) |  | -5.887 (-6.886, -4.888) |  |
| Hb (g/L) |  |  |  |  |  |  |  |
| Q1 | 90 | Ref. | 0.226 | Ref. | <0.001 | Ref. | 0.391 |
| Q2 | 69 | -0.163 (-0.426, -0.101) |  | -0.055 (-0.084, -0.026) |  | -0.558 (-1.835, 0.720) |  |
| Q3 | 69 | -0.481 (-0.737, -0.226) |  | -0.069 (-0.098, -0.041) |  | -2.294 (-3.532, -1.055) |  |
| Q4 | 70 | -0.590 (-0.859, -0.321) |  | -0.078 (-0.108, -0.049) |  | -2.496 (-3.80, -1.192) |  |

Hb: hemoglobin, SF: serum ferritin, IPL: interpeak latency, SES: socioeconomic status.

**Supplementary Table 8 Associations between neonatal SF, maternal Hb and neonatal absolute latency wave** V**, IPL** III-V**, and SP/AP ratio (wave** V**) in moderate SES.**

| Iron  status | Moderate SES | | Absolute latency wave V |  | IPL III-V |  | SP/AP ratio |  |
| --- | --- | --- | --- | --- | --- | --- | --- | --- |
|  | *n* | | *β* (95% CI) | *P* | *β* (95% CI) | *P* | *β* (95% CI) | *P* |
| SF (ng/mL) | |  |  |  |  |  |  |  |
| Q1 | | 51 | Ref. | <0.001 | Ref. | <0.001 | Ref. | <0.001 |
| Q2 | | 73 | -0.865 (-1.118, -0.612) |  | -0.077 (-0.103, -0.052) |  | -3.984 (-5.252, -2.716) |  |
| Q3 | | 89 | -0.870 (-1.114, -0.627) |  | -0.099 (-0.123, -0.074) |  | -4.271 (-5.492, -3.051) |  |
| Q4 | | 91 | -0.980 (-1.223, -0.738) |  | -0.108 (-0.132, -0.083) |  | -5.437 (-6.653, -4.222) |  |
| Hb (g/L) | |  |  |  |  |  |  |  |
| Q1 | | 51 | Ref. | 0.155 | Ref. | 0.141 | Ref. | 0.021 |
| Q2 | | 73 | -0.188 (-0.447, 0.071) |  | -0.020 (-0.046, 0.007) |  | -1.543 (-2.847, -0.239) |  |
| Q3 | | 89 | -0.442 (-0.694, -0.190) |  | -0.039 (-0.065, -0.013) |  | -2.804 (-4071, -1.537) |  |
| Q4 | | 91 | -0.378 (-0.634, -0.121) |  | -0.050 (-0.076, -0.023) |  | -1.809 (-3.098, -0.519) |  |

Hb: hemoglobin, SF: serum ferritin, IPL: interpeak latency, SES: socioeconomic status.

**Supplementary Table 9 Associations between neonatal absolute latency wave** V**, IPL** III-V**, and SP/AP ratio (wave** V**) in high SES**

| Iron  status | High SES | | Absolute latency wave V |  | IPL III-V |  | SP/AP ratio |  |
| --- | --- | --- | --- | --- | --- | --- | --- | --- |
|  | *n* | | *β* (95% CI) | *P* | *β* (95% CI) | *P* | *β* (95% CI) | *P* |
| SF (ng/mL) | |  |  |  |  |  |  |  |
| Q1 | | 75 | Ref. | <0.001 | Ref. | <0.001 | Ref. | <0.001 |
| Q2 | | 69 | -0.800 (-1.048, -0.552) |  | -0.076 (-0.102, -0.049) |  | -4.618 (-5.954, -3.282) |  |
| Q3 | | 58 | -0.747 (-1.007, -0.487) |  | -0.091(-0.119, -0.064) |  | -6.766 (-8.166, -5.365) |  |
| Q4 | | 55 | -0.928 (-1.192, -0.665) |  | -0.109(-0.137, -0.081) |  | -6.552(-7.974, -5.130) |  |
| Hb (g/L) | |  |  |  |  |  |  |  |
| Q1 | | 75 | Ref. | 0.01 | Ref. | 0.248 | Ref. | 0.284 |
| Q2 | | 69 | -0.333 (-0.587, -0.079) |  | -0.016 (-0.043, -0.011) |  | -0.802 (-2.272, 0.668) |  |
| Q3 | | 58 | -0.404 (-683, -0.125) |  | -0.040(-0.070, -0.010) |  | -3.107(-4.724, -1.491) |  |
| Q4 | | 55 | -0.580 (-0.886, -0.275) |  | -0.055(-0.088, -0.022) |  | -3.458(-5.225, -1.691) |  |

Hb: hemoglobin, SF: serum ferritin, IPL: interpeak latency, SES: socioeconomic status.

**Supplementary Table 10 Mediation analyses of the associations of SES with auditory neural maturation [absolute latency wave V, IPL III-V, and SP/AP ratio] by ID**

| Variables | Absolute latency wave V | IPL III-V | SP/AP ratio |
| --- | --- | --- | --- |
|  | *β* (SE) *P*-value OR (95% CI) | *β* (SE) *P*-value OR (95% CI) | *β* (SE) *P*-value OR (95% CI) |
| SES level |  |  |  |
| Total effect-path c^1^ | 0.0121 (0.0347) 0.7285 1.012(95%CI:0.945,1.084) | 0.0009 (0.0212) 0.9668 1.001(95%CI:0.960,1.043) | 0.0968(0.1821) 0.5953 1.102 (95%CI:0.771,1.575) |
| Path a^1^ | -0.0097 (0.0477) 0.8384 | -0.0097 (0.0477) 0.8384 | -0.0097(0.0477) 0.8384 |
| Path b^2^ | -0.2722 (0.0231) <0.001 | -0.0099 (0.0006) <0.001 | -1.9191 (0.1128) <0.001 |
| Direct effect-path c´^2^ | 0.0094 (0.0322) 0.7704 0.910 (95%CI: 0.948,2.069) | -0.0010 (0.0191) 0.9577 0.999 (95%CI: 0.962,1.037) | 0.0781 (0.1575) 0.6202 1.081 (95%CI:0.794,1.473) |
| Indirect effect | 0.0026 (0.0132) 0.7910 1.003 (95%CI: 0.977,1.029) | 0.0019 (0.0091) 0.9465 1.002 (95%CI: 0.985,1.021) | 0.0187 (0.0911) 0.5154 1.052 (95%CI: 0.851, 1.215) |
| Proportion (%) ^#^ | 21.82 | 10.67 | 19.23 |

^1^ Adjusted for mother (age, gestational age, Hb, FBG, BMI, extra-sport activities, hypertension, smoking, drinking, dietary pattern, cesarean delivery, assisted reproductive technology) and

newborn (birth weight, Apgar score, gender)

^2^ Adjusted for mother (age, gestational age, Hb, FBG, BMI, extra-sport activities, hypertension, smoking, drinking, dietary pattern, cesarean delivery, assisted reproductive technology) and

newborn (birth weight, Apgar score, gender, heel prick blood SF)

^#^ Mediation effect percentage = (a × b)/c×100%

SES, socioeconomic status, ID: iron deficiency, AP: action potential, SP: summating potential, IPL: interpeak latency, FBG: fasting blood glucose, BMI: body mass index, Hb: hemoglobin.

**Supplementary Table 11 Combined effects of iron status and SES on auditory neural maturation [absolute latency wave V, IPL III-V, and SP/AP ratio] and children OME ^a^**

| Variables | OME | | High wave V (> 50%) | | Wave Ⅴ | | High IPL III-V (> 50%) | | IPL III-V | | High SP/AP ratio (> 50%,) | | SP/AP ratio | |
| --- | --- | --- | --- | --- | --- | --- | --- | --- | --- | --- | --- | --- | --- | --- |
|  | OR (95% CI) | *P* | OR (95% CI) | *P* | *β* (95% CI) | *P* | OR (95% CI) | *P* | *β* (95% CI) | *P* | OR (95% CI) | *P* | *β* (95% CI) | *P* |
| High SES + IS | Reference |  | Reference |  | Reference |  | Reference |  | Reference |  | Reference |  | Reference |  |
| High SES + ID | 1.176 (0.824, 1.679) | 0.081 | 2.117 (1.147, 3.905) | 0.016 | 0.254 (0.010, 0.498) | 0.042 | 1.544 (0.893, 2.668) | 0.120 | 0.185 (0.043, 0.318) | 0.003 | 2.184 (1.254, 3.805) | 0.006 | 0.132 (0.011, 0.775) | 0.034 |
| Moderate SES + IS | 0.629 (0.304, 1.302) | 0.212 | 0.905 (0.613, 1.334) | 0.612 | 0.010 (-0.104, 0.124) | 0.864 | 0.955 (0.501, 1.821) | 0.889 | -0.015 (-0.074, 0.043) | 0.858 | 0.729 (0.380, 1.389) | 0.333 | 0.027 (-0.583,0.637 ) | 0.739 |
| Moderate SES + ID | 0.983 (0.658, 1.975) | 0.201 | 1.866 (1.087, 3.195) | 0.024 | 0.664 (-0.032, 0.109) | 0.507 | 1.367 (0.802, 2.328) | 0.250 | 0.083 (0.054, 0.132) | 0.154 | 1.381 (0.805, 2.370) | 0.241 | 0.084 (-0.278, 0.446) | 0.150 |
| Low SES + IS | 0.791 (0.389, 1.608) | 0.517 | 1.438 (0.696, 2.969) | 0.326 | -0.035 (-0.102, -0.076) | 0.699 | 1.694 (0.389, 1.608) | 0.146 | 0.126 (0.091, 0.160) | 0.159 | 1.385 (0.679, 2.827) | 0.370 | -0.030 (-0.382, 0.322) | 0.740 |
| Low SES + ID | 1.624 (1.133, 3.667) | 0.037 | 2.448 (1.304, 4.598) | 0.005 | 0.104 (0.058, 0.143) | 0.045 | 1.366 (0.806, 2.315) | 0.246 | 0.124 (0.097, 0.151) | 0.027 | 1.787 (1.047, 3.049) | 0.033 | 0.108 (-0.105, 0.320) | 0.055 |

^a^ Adjusted for mother age, gestational age, Hb, FBG, BMI, extra-sport activities, hypertension, smoking, drinking, dietary pattern, cesarean delivery, assisted reproductive technology) and newborn (birth weight, Apgar score, gender, heel prick blood SF).

SES, socioeconomic status, ID: iron deficiency, AP: action potential, SP: summating potential, IPL: interpeak latency, FBG: fasting blood glucose, BMI: body mass index, Hb: hemoglobin.

**Supplementary Table 12 Associations of Iron status and SES with OME prevalence after excluded children with adenoid hypertrophy, chronic sinusitis, and allergic rhinitis.**

| Variables | Model 1 | |  | Model 2A | |  | Model 2B | |
| --- | --- | --- | --- | --- | --- | --- | --- | --- |
|  | OR (95 % CI) | *P*-value |  | OR (95 % CI) | *P*-value |  | OR (95 % CI) | *P*-value |
| Iron status |  |  |  |  |  |  |  |  |
| IS | Reference |  |  | Reference |  |  |  |  |
| ID | 3.093 (1.302, 7.347) | 0.011 |  | 3.195 (1.346, 7.634) | 0.009 |  |  |  |
| Socioeconomic status |  |  |  |  |  |  |  |  |
| High | Reference |  |  |  |  |  | Reference |  |
| Moderate | 0.946 (0.495, 1.808) | 0.433 |  |  |  |  | 0.753 (0.384, 1.476) | 0.409 |
| Low | 1.307 (0.669, 2.555) | 0.867 |  |  |  |  | 1.097 (0.570, 1.711) | 0.781 |

Model 1: crude model, no adjustment.

Model 2A: adjusted for mother (age, gestational age, FBG, BMI, extra-sport activities, hypertension, smoking, drinking, dietary pattern, cesarean delivery, assisted reproductive technology)

newborn (birth weight, Apgar score, gender), SES.

Model 2B: adjusted for mother (age, gestational age, FBG, BMI, extra-sport activities, hypertension, smoking, drinking, dietary pattern, cesarean delivery, assisted reproductive technology, Hb)

newborn (birth weight, Apgar score, gender, heel prick blood SF).

OR: odds ratios, CI: confidence intervals, SES: socioeconomic status, OME: otitis media with effusion, FBG: fasting blood glucose, BMI: body mass index, Hb: hemoglobin.

**Supplementary Table 13 Sensitivity analyses by additionally adjusted for more COVID-19 Symptoms**

|  | Total |  | Low SES |  | Moderate SES |  | High SES |  |
| --- | --- | --- | --- | --- | --- | --- | --- | --- |
|  | OR (95 % CI) |  | OR (95 % CI) |  | OR (95 % CI) |  | OR (95 % CI) |  |
| Iron status | OME prevalence | *P* | OME prevalence | *P* | OME prevalence | *P* | OME prevalence | *P* |
| Model 1^*^ |  |  |  |  |  |  |  |  |
| IS | Reference |  | Reference |  | Reference |  | Reference |  |
| ID | 3.300 (1.379, 7.874) | 0.007 | 6.622 (1.567, 13.778) | 0.010 | 2.577 (0.929, 7.143) | 0.069 | 2.874 (1.085, 7.634) | 0.034 |
| Model 2^*^ |  |  |  |  |  |  |  |  |
| IS | Reference |  | Reference |  | Reference |  | Reference |  |
| ID | 2.851(1.192, 6.821) | 0.019 | 6.647 (1.547, 18.560) | 0.011 | 2.296 (0.881, 5.406) | 0.068 | 2.778 (0.876, 7.428) | 0.067 |

^*^ Adjusted for mother (age, gestational age, Hb, FBG, BMI, extra-sport activities, hypertension, smoking, drinking, dietary pattern, cesarean delivery, assisted reproductive technology), newborn (birth weight, Apgar score, gender)

^**^ Adjusted for for mother (age, gestational age, Hb, FBG, BMI, extra-sport activities, hypertension, smoking, drinking, dietary pattern, cesarean delivery, assisted reproductive technology), newborn (Birth weight, Apgar score, gender), and COVID-19 Symptoms (fever, sore throat, earache, hearing loss, ear tightness, decreased sense of smell, nasal congestion, fatigue, muscle soreness, cough).

OR: odds ratios, CI: confidence intervals, SES: socioeconomic status, OME: otitis media with effusion, FBG: fasting blood glucose, BMI: body mass index, Hb: hemoglobin.
